# Enhancing end-of-life care in Ghana: nurse strategies and practices in addressing patient needs

**DOI:** 10.1186/s12904-025-01706-5

**Published:** 2025-03-14

**Authors:** Evans Appiah Osei, Agnes Abena Aquah, Stella Appiah, Lalani Nasreen, Jenifer Oware, Collins Sarpong, Zachariah Asuboni, Hillary Ongoyo Omoze

**Affiliations:** 1 Department of Nursing/Gerontology, Purdue University, 425 South River Road, West Lafayette, Indiana USA; 2 Department of Nursing, Valley View University, Accra, Ghana; 3 Niagara College, Palliative Care– Multidiscipline, Welland, Canada; 4 Korlebu Teaching Hospital, Ghana Health Service, Accra, Ghana; 5 Department of Nursing, University of Ghana, Accra, Ghana; 6 Department of Education, Purdue University, West Lafayette , Indiana USA

**Keywords:** End of life, Experiences, Hospitals, Nurses, Perceptions, Strategies

## Abstract

**Background:**

Globally, end-of-life care is vital for individuals in their final months or years, emphasizing comfort and dignity. However, the provision of palliative care in low-resource countries, such as Ghana, remains inadequately documented and poorly understood. This study aimed to identify the specific end-of-life care needs of patients and families and explore strategies to enhance end of life care practices among nurses in selected settings in Accra, Ghana.

**Methodology:**

This qualitative research utilized in-depth, one-on-one interviews using semi-structured interviews in a sample of *N* = 32 nurses working in two selected hospitals in Ghana. Thematic content analysis was used to analyze the data. Participants were purposively selected, with the sample size determined by data saturation.

**Results:**

The analysis identified three main themes and eleven subthemes. The main themes were: providing dignity and comfort care, respecting ethical values, and perceptions of end-of-life care. The subthemes included: showing presence, demonstrating compassion, addressing challenges in end-of-life conversations, fostering autonomy and respect, managing gratitude and discontent, helping patients accept their condition, seeking additional training, building emotional connections, involving families, and respecting patients’ cultural, social, and religious beliefs. The participants had cared for or were currently caring for patients aged 50–75 years with cancer, organ failures, Advanced Heart diseases and cognitive disorders. Participants described their efforts to make the last days of their patients and families memorable as possible.

**Conclusions:**

Nurses in Ghana provide compassionate care, addressing pain relief, ethical concerns, and patient expressions of gratitude and discontent, with their efforts influenced by religious and cultural factors. To enhance end of life care quality, policymakers should implement structured end-of-life care training for nurses and develop culturally aligned palliative care guidelines to meet the needs of patients receiving end of life care.

**What is Already Known on This Topic**

- The meaning and components of hospice/end-of-life care.
- The significance of end-of-life care for patients, families, and the palliative care team.
- Resource and personnel shortages affecting end-of-life care provision in Africa.

**What This Study Adds**

- Strategies to enhance end-of-life care for patients.
- Firsthand accounts of nurses providing end-of-life care in a low-resource country.
- Insights into how nurses navigate ethical issues while providing end-of-life care.

**How This Study Might Affect Research**,** Practice**,** or Policy**.

- The findings highlight the need for support not only for patients and families in hospice but also for care providers.
- The study will aid in developing effective interventions to make the final days of patients more memorable and comfortable.

## Introduction

The significance of end-of-life (EOL) care is increasingly recognized globally due to its profound impact on the well-being of patients and their families during the final stages of life [1, 2]. EOL care is a vital component of healthcare, aimed at providing comfort, dignity, spiritual care and quality of life to patients nearing the end of their lives [3, 4]. This care involves managing physical symptoms, offering psychological support, and providing spiritual and emotional care to both patients and their families [5, 6].

Despite the recognized importance of end-of-life (EOL) care, a significant gap exists in the literature concerning the specific strategies and practices employed by nurses to address the diverse needs of patients in low-resource countries, particularly Ghana [7]. Most existing research focuses on EOL care in high-income countries [8, 9], which leaves a substantial gap in understanding the unique challenges and practices in culturally diverse and resource-constrained environments.

Cultural and religious beliefs significantly influence end-of-life perceptions and care in Africa, including Ghana. In many African cultures, becoming an ancestor after death is considered an honorable goal. However, practices such as advance care directives are often avoided, as they are believed to undermine this honor [10]. In Tanzania, South Africa, and Kenya, diseases like HIV/AIDS are stigmatized and viewed as resulting from immoral behavior or poor lifestyle choices, which can negatively impact the care and treatment provided at the end of life [11–13]. Studies in Kenya and Tanzania indicate that individuals dying from such “bad diseases” are often excluded from ancestral recognition. In Ghana, death is perceived as a peaceful visitor that brings rest, yet culturally, those who die young are considered to have had a “bad death” and may not receive proper funeral rites [14, 15]. Additionally, many religious patients in Africa rely on faith for healing, often foregoing medical treatment in favor of divine intervention [16].

Ghana’s healthcare system faces numerous challenges, including a decline in insurance coverage, with approximately 40% of patients uninsured, and delayed NHIS claim payments exceeding nine months [17, 18]. These issues contribute to nearly half of the nurses in Ghana considering emigration to developed countries for better opportunities [19]. The nurse-to-patient ratio is inadequately low compared to other nations [18]. Research highlights challenges in palliative care delivery, such as distress over patient mortality, difficulties in communicating bad news, and frustration with resource shortages [20]. Additionally, there is a reported shortage of palliative care staff in Ghana [21].

While substantial research exists on end-of-life (EOL) care in developed countries, the contextual challenges and innovative practices in settings like Ghana remain underexplored [22]. Understanding these aspects is crucial for developing tailored interventions that improve EOL care in similar environments [23]. Our study therefore identified the specific end-of-life care needs of patients and families while exploring strategies to improve end-of-life care practices among nurses in selected settings in Accra, Ghana.

.

## Methods

### Study design

The study utilized a descriptive phenomenological design to examine the specific end-of-life care needs of patients and families and to explore strategies for improving end-of-life care practices among nurses in selected hospitals in Accra, Ghana. Descriptive phenomenology is well-suited for exploring lived experiences without preconceived notions, as it focuses on understanding the essence of particular experiences [24, 25]. This approach provides insight into the lived meanings of experiences and is especially useful for investigating new areas with limited theoretical foundations [24, 26]. Furthermore, it is appropriate for examining contextual factors unique to the population under study [27]. This method is ideal for gaining a deeper understanding of the subjective experiences and meanings nurses ascribe to their roles in end-of-life care.

### Setting

The study was conducted in two selected hospitals in Accra, Ghana, specifically chosen for their well-established palliative care units and diverse patient demographics.

### Sampling technique and eligibility criteria

Participants were selected through purposive sampling, a technique well-suited for identifying information-rich cases relevant to the phenomenon of interest and capable of providing insights into specific themes or concepts [28, 29]. This method allowed the researchers, particularly the second author, to recruit nurses from the selected facilities who had cared for patients with life-limiting conditions such as cancer, end-stage renal failure, and advanced chronic heart conditions. Eligibility criteria required participants to be nurses from the two selected hospitals in Accra who had experience caring for patients with chronic conditions at the end of life. Nurses who declined participation or were busy with nursing task during the recruitment period were excluded from the study. Other palliative care team members, such as physicians, pharmacists, social workers, and psychologists, were excluded as the study aimed to focus on the unique roles, and contributions of nurses as primary caregivers.

### Sampling size

The sample size was determined based on data saturation, which occurred when no new information or insights emerged from participants regarding strategies to enhance end-of-life care, prompting the conclusion of data collection. Initially, 50 nurses were approached for the study, with 35 agreeing to participate (15 from one facility and 20 from the other). Of these, data collection concluded after interviewing 32 participants (15 from the first facility and 17 from the second, which had a higher patient load). Redundancy in responses began with the 30th participant, and two additional interviews were conducted to confirm saturation. The remaining three nurses from one of the facilities who had agreed to participate were informed of the study’s closure due to saturation, which they accepted. The final sample comprised 32 participants, including 10 males and 22 females.

Ethical approval was obtained from the Dodowa institutional review board (IRB), and all participants provided informed verbal and written consent, ensuring autonomy and voluntary participation.

### Data collection

Data were collected through face-to-face interviews using a semi-structured interview guide developed by the researchers and reviewed by nursing experts. The guide included open-ended questions and probes regarding nurses’ experiences in EOL care. It consisted of 3 main questions and 9 probes,. The three main questions focused on EOL care experiences, perceptions, and strategies for addressing patients’ needs.

Participants were recruited by the second author following approval from the two facilities to approach eligible nurses. The recruitment process involved multiple visits to each facility to identify nurses who met the inclusion criteria and were willing to participate. A total of 20 nurses from one hospital and 15 from the other consented to join the study. Recruitment primarily took place in the nurses’ stations during their breaks, with support and facilitation from the ward in-charges. Additionally, a few ward in-charges reached out to eligible nurses who were absent, and these nurses were later approached to secure their consent for participation.

The interviews took place in private spaces within hospitals and participants’ homes to ensure privacy and comfort. Each interview lasted approximately 30–40 min. Data collection commenced in January 2024 and concluded in April 2024. Participants consented to audio recording of the interviews, supplemented by field notes. Before the interviews, AA introduced herself and explained the study’s purpose. The interview guide underwent pilot testing with 6 nurses to refine questions, ensure clarity, and address uncertainties prior to the main interviews. Data security measures included anonymizing transcripts and securely storing digital files.

### Data analysis

The data underwent inductive content analysis using an iterative process [19]. This method, often applied in phenomenological research, encourages an open-minded examination of the data, allowing themes and patterns to emerge organically from participants’ lived experiences, free from pre-existing theories or categories [30, 31]. Audio recordings were transcribed verbatim and reviewed for accuracy by all authors. The initial analysis was conducted collaboratively by the first three authors, who engaged in multiple readings of the transcripts to familiarize themselves with the data. They categorized meaning units, coded significant statements, and grouped codes into broader themes [32]. Subsequently, the analysis was further reviewed by the remaining five authors. Regular debriefing sessions were held among the research team to discuss coding decisions and ensure consistency throughout the analysis process.

### Trustworthiness

To enhance the trustworthiness of the study, we implemented strategies such as member checking, peer debriefing, and maintaining an audit trail. The study underwent peer review by all authors and colleague researchers to gather valuable feedback. Additionally, the interview guide was piloted with six participants to refine and improve its structure and content. Peer debriefing sessions facilitated in-depth discussions among the authors on data coding and interpretation, ensuring agreement on the identified codes. While the initial coding was conducted by the first three authors, the final codes were thoroughly reviewed and unanimously approved by all authors, ensuring the credibility and rigor of the findings. Also, the last two authors reviewed and agreed upon themes and subthemes. Additionally, three researchers had prior experience with EOL studies and patient care. A reflexive journal was kept to maintain reflexivity and reduce bias, where one author reflected on personal experiences, including caring for her grandfather during his final stages of a severe stroke, discussing emotional and financial challenges. Authors discussed these experiences before data collection to mitigate their influence on results. To maintain anonymity, participants were assigned pseudonyms based on their rank, respondent number, and years of experience. For example, a Senior Nursing Officer was labeled as “SNO.” Additional ranks and their corresponding labels are provided in Table 1. Each participant’s interview order was also indicated, with the 10th respondent represented as “R10,” along with their years of experience.

## Results

### Socio-Demographic characteristics

The majority of participants were aged 18–29 years (65.6%), followed by those aged 30–49 years (28.1%), with a smaller proportion aged 50 years and above (6.3%). Additionally, females comprised a larger portion of the sample (68.75%) compared to males (31.25%). For detailed socio-demographic data, refer to Table 1.

.

**Table 1:** Socio-demographic characteristics of participants

Variable			Frequency *N* = 32			Percentage 100%	
Age 18–29 years 30–49 years 50 years and above			21 9 2			65.6 28.1 6.3	
Gender Males Females			10 22			31.25 68.75	
Marital status Single Married			20 12			62.5 37.5	
Religion Christian Muslim Other		15 10 7			46.9 31.2 21.9		
Location Osu Mamprobi Nima Madina Pokuasi Teshi Labadi East legon			6 4 5 5 2 3 2 5			18.75 12.5 15.6 15.6 6.2 9.4 6.3 15.6	
Qualification Diploma Degree Masters			5 12 15			15.6 37.5 46.9	
Years of experience 1–5 5–10 10–15 15–20			8 10 10 4			25 31.3 31.3 12.5	
Rank Senior nursing officer (SNO) Nursing officer (NO) Senior staff nurse (SSN) Staff nurse (SN)			10 8 10 4			31.3 25 31.3 12.5	
Have expertise in palliative care Yes No			15 17			46.9 53.1	
Conditions managed Cancer Organ failure Chronic heart diseases Neurodegenerative diseases AGE of patients 50–59 60–69 70 and above			13 13 4 2 10 8 14			40.6 40.6 12.5 6.3 31.25 25 43.75	

Source; interview data,2024

### Organization of themes

Three themes with 11 sub themes emerged from the data analyzed and presented in Table 2.

**Table 2:** Themes and subthemes

THEMES	SUBTHEMES
Theme 1 Provision of dignity and comfort care Theme 2 Respecting Ethical Values	Sub-theme 1.1 Showing Presence, Dignity and Comfort in Care Sub-theme 1.2 Compassion for complete pain and symptom relief Sub-theme 1.3 Tackling Challenges in End-of-Life Conversations Sub-theme 2.1 Autonomy and Respect Sub-theme 2.2 Managing Patient Mixed Reactions: Gratitude and Discontent Sub-theme 2.3 Assisting Patients in Accepting Their Condition’s Reality
Theme 3 Perceptions about End-of-life Care Delivery	Sub-theme 3.1 Aspiring for Additional Training in End-of-Life Care Sub-theme 3.2 Perceiving End-of-Life as Inevitable and Natural Sub-theme 3.3 Nurses’ Views on Family Engagement in End-of-Life Care Sub-theme 3.4 Respecting Patients’ Cultural, Social, and Religious Beliefs Sub-theme 3.5 Managing Emotional Connections with Patients

### Theme 1: provision of dignity and comfort care

This theme highlights the critical role of nurses in ensuring patients experience respect, relief, and emotional support during end-of-life care. This encompasses showing presence and dignity, demonstrating compassion to alleviate pain and symptoms, and addressing the challenges of navigating sensitive end-of-life conversations.

#### Sub-theme 1.1 showing presence, dignity and comfort in care

Creating a supportive environment through enhancing presence is vital in end-of-life care, where emotional connection significantly impacts patients’ well-being. Participants emphasized ensuring family presence to enhance communication and shared connections, as reflected in the quote below.

.:

*“Unlike other patients with other conditions where families visit only during visiting times*,* for patients receiving end of life here we ensure family are available 24 hours by running shifts*,* because we always want them to be able to share their problems with their love ones and always engage in communication to help them not to think too much*,* and we have observed that patients love it when they are chatting with their families especially those with partners and children. We sometimes see them sing and watch television together and we allow them enough time to do that”***(SNO**,** R11**,** 5 years of experience)**.

Dignity and comfort were enhanced by celebrating special occasions like birthdays and anniversaries for patients nearing the end of life. This practice demonstrated respect for their humanity, acknowledged the significance of their lives, and created joyful, shared moments with loved ones. As one participant explained:

*“Celebrating special occasions such as birthdays and anniversaries plays a crucial role in bringing joy and fostering connections between patients and their families. For example*,* when patients are informed that they may have less than a year to live*,* we help family members understand the situation. Together*,* we organize significant birthday celebrations*,* inviting friends and church members*,* recognizing that it could be the patient’s last birthdays****(SN***,*** R10***,*** 5 years of experience)***.

Some also highlighted the nurse’s commitment to creating lasting, positive memories for patients and their families by:

*“Patients who are mobile and whose conditions allow are occasionally permitted to go on outings with their families to locations such as the beach or other recreational centers of their choice. They are required to sign out when leaving and sign in upon return*,* and are provided with contact information for use in case of necessity. Our goal is to make their lives as normal and memorable as possible”*. **(SNO**,** R13**,** 6 years of experience)**

Creating an environment where patients feel valued and heard contributes significantly to their well-being. Nurses strive to ensure that patients have opportunities to engage in activities that are meaningful to them.

*“Sometimes*,* it’s as simple as arranging for a patient to have their favorite meal or listen to their favorite music. These small gestures make a big difference in their quality of life”*. **(SN**,** R12**,** 3 years of experience)**

These practices highlight the commitment to fostering dignity and comfort by creating meaningful connections through family involvement, celebrating birthdays, and facilitating patients’ favorite meals and music, leaving lasting memories during end-of-life care.

#### Sub-theme 1.2 compassion for complete pain and symptom relief

Nurses offered compassion and adopted various approaches to relieve total pain and symptom management providing end-of-life care. Using a holistic care approach, nurses were responsive to managing pain through medication, providing psychological support, and ensuring physical comfort. thus, enhancing the overall quality of life for patients.

*“Managing symptoms effectively is crucial for their time left. Pain medications are not delayed as most of them have severe pain. We use a combination of medications prescribed to improve their pain as well as their sleep”***(NO**,** R13**,**7 years of experience)**.

*“In certain circumstances*,* we communicate with the doctor to recommend therapy sessions*,* particularly for individuals struggling with ineffective coping mechanisms or experiencing profound sadness. These individuals are referred to a psychotherapist for sessions aimed at alleviating symptoms and enhancing coping skills”***(NO**,** R14**,**7 years of experience)**.

*“Sometimes all they need to be ok and for them to forget about their problems is the time we spend with them and our readiness to listen to their concerns*,* so we do not joke with this. We spend a lot of time talking to patients*,* understanding their fears*,* and reassuring them”.***(SN**,** R11**,** 5 years of experience)**

Effective symptom management often requires a multidisciplinary approach, involving collaboration with other health care professionals to address all aspects of patient care.

*“Some of them have several problems (Like one patient is to more than 3 conditions) which cannot be solved by just the physician and the nurses unlike the general med/surg wards. We need everyone to help to make them feel better. We work closely with doctors*,* physiotherapists*,* and counselors to create a comprehensive care plan that addresses the physical and emotional needs of our patients”*. **(SSN**,** R10**,** 8 years of experience)**

In conclusion, effective symptom management in end-of-life care relies on a multidisciplinary approach that prioritizes timely pain relief, emotional support, and collaborative efforts with other healthcare professionals to address the holistic needs of patients.

#### Sub-theme: 1.3 tackling challenges in End-of-Life conversations

End-of-life conversations are crucial but challenging aspects of nursing care. These discussions require sensitivity, honesty, and the ability to provide comfort and reassurance to patients and their families.

*“Discussing end-of-life care is never easy. One difficult conversation I had was with a patient with end-stage cancer who asked*,* ‘How much time do I have left?’ His wife broke down*,* saying*,* ‘I can’t bear to lose him.’ Sometimes they keep asking several questions when you have not even began answering one. It is sometimes challenging”*. **(NO**,** R30**,** 7 years of experience)**

*“In various instances*,* as we endeavor to engage patients in dialogue*,* they challenge us with questions like*,* ‘Have you ever undergone renal failure?’ or make statements like ‘You don’t understand my situation.’ Such statements can leave us speechless at times”* (SSN, R20,12 years of experience).

Some nurses expressed difficulty in being completely transparent with patients due to their health condition.

*“We frequently encounter challenging questions*,* such as when patients*,* who have been informed of their limited life expectancy*,* ask*,* ‘Am I going to die soon?’ This raises concerns about whether they comprehend the information or are in denial of reality”. (SN*,* R21 4 years of experience*) **.**

Effective communication strategies and continuous professional development can enhance nurses’ skills in handling these difficult conversations.

*“Incorporating communication skills training into the nursing curriculum is essential to address this issue. Additionally*,* regular debriefing sessions with colleagues can significantly enhance our ability to navigate end-of-life discussions with patients and their families. These sessions provide opportunities for reflection on challenging conversations*,* facilitate the sharing of experiences*,* and offer valuable support”.* (SSN, R18, 10 years of experience)

In conclusion, navigating end-of-life discussions requires enhanced communication skills, emotional resilience, and continuous professional development to support both patients and families during these profoundly challenging moments.

### Theme 2: respecting ethical values

The nurses disclosed that while providing care, they had to uphold the ethical values of their patients by carefully handling issues of autonomy and respect, managing both gratitude and resentment from patients, and sensitively guiding patients towards accepting death and dying.

#### Sub-theme 2.1 autonomy and respect

Nurses often face ethical dilemmas in balancing patient autonomy with what they believe to be in the patients’ best interests. This involves respecting patients’ wishes and making decisions that align with their values and preferences, even when these decisions may conflict with medical advice.

*“Respecting a patient’s autonomy is challenging*,* especially when their choices might lead to a less favorable outcome. For example*,* a 68-year-old patient with advanced prostate cancer chooses watchful waiting over surgery because he thought his time is due and does not want any medical treatment stress*,* despite medical advice. Even though we had to ensure his comfort and the surgery was one of the treatments*,* there was nothing we health providers could do aside the pain medications”*. **(SNO**,** R16**,** 10 years of experience)**

*“It’s difficult when a patient’s decision conflicts with medical advice. We have to ensure that they are fully informed and that their choices are respected*,* even if it’s not what we would recommend*,* sometimes we allow them time to discuss with their families to come to agreement on what is the best option for them*,* we provide information where necessary so that they know the pros and cons*,* but this sometimes can take forever and we wish we could go ahead with the treatment to make them better*,* but due to ethical reasons*,* we have to wait”***(NO**,** R11**,** 7 years of experience)**.

Balancing these ethical considerations often requires in-depth discussions and a compassionate approach to understand and honor the patient’s perspective.

*“We must strongly advocate for the patient’s desires*,* even if they diverge from medical interventions. It’s about respecting their autonomy while ensuring they are fully informed about potential consequences. There have been instances where patients were not adequately informed*,* prompting us to advocate by reaching out to relevant stakeholders to ensure they receive comprehensive information for making informed decisions”.***(SSN**,** R20**,**12 years of experience)**

In conclusion, respecting patient autonomy in end-of-life care requires balancing ethical principles, providing comprehensive information, and fostering compassionate dialogue to support patients in making informed decisions that align with their values and preferences.

#### Sub-theme 2:2 managing patient mixed reactions: gratitude and discontent

Nurses frequently encounter mixed reactions from patients and their families, ranging from gratitude to discontent. Managing these reactions requires empathy, patience, and effective communication skills to provide the best possible care and support.

*“We frequently receive sincere expressions of gratitude from patients and their families for the care and support we provide. This is particularly prevalent among terminally ill patients who have reached the acceptance stage of their grieving process and have found inner peace. When we approach their bedside to administer care*,* they often verbalize their appreciation and extend blessings for the kindness we’ve shown them (God bless you). For me*,* there’s no greater source of encouragement than this; it surpasses the value of money or any material gift”*. **(SN**,** R25**,**6 years of experience)**

*“A patient in end-stage renal failure expressed these sentiments to us: “If we hadn’t met you*,* how would we have coped? You’ve shown us that you’re like family*,* caring deeply about our needs.” Such a heartwarming and soul-lifting message. What is more than this?”***(SN**,** R 27**,**6 years of experience)**.

While expressions of gratitude are frequent, certain families may experience dissatisfaction stemming from the inevitable decline of their loved ones, leading to concerns among health care providers.

*“At times*,* despite our best efforts*,* some families feel dissatisfied due to the inevitable decline of their loved one’s health. For example*,* a patient’s daughter once expressed*,* ‘I feel like nothing is improving*,* and we’re just watching him fade away.’ ‘You always assure me it is going to be well but I am not improving*,* it is like you guys are deceiving me and not been fair to me’ At times*,* it becomes challenging for us*,* but we are here to provide care”.*

**(NO**,** R15**,**5 years of experience).**

**“***A patient once expressed*,* ‘I was doing better while at home. Despite the money spent and the treatment received*,* my condition is worsening. It feels like you’re not giving your best. I trusted you all to ensure I feel better*,* but it seems otherwise. With words like this you go to your bed and you keep think about it”.***(NO**,** R30**,** 7 years of experience)**

While heartfelt expressions of gratitude from patients and families serve as a profound source of encouragement for nurses, challenges arise when unmet expectations or the inevitable progression of illness lead to dissatisfaction, highlighting the emotional complexities of providing end-of-life care.

#### Sub-theme 2.3 promoting acceptance of death and dying

Helping patients come to terms with their condition is a significant part of end-of-life care. Nurses support patients through counseling, emotional support, and fostering an environment where patients feel safe to express their feelings.

*“We empathize with patients who struggle to accept the reality of their condition as their life approaches its end. Our approach involves offering ongoing support*,* providing necessary information*,* and lending a compassionate ear to their concerns. We ensure they have access to resources such as counseling*,* allowing them to express their emotions openly”*. **(NO**,** R31**,** 6 years of experience)**

*“We spend not less than 2–3 hours daily talking to patients and giving them some hope. Encouraging patients to talk about their fears and hopes and concerns can be therapeutic*,* helping them to find peace and acceptance in their situation”*. **(SN**,** R30 **,**5 years of experience)**

Building a trusting relationship with patients is essential in this process, as it enables them to feel secure and supported in their journey.

*“Trust and rapport are crucial. We gain their trust by providing them with full information about their condition and giving honest answers to their questions. We also make their needs a priority to gain their trust. Initially when they are admitted you find them to be a bit reserved but after 2days to a week when they begin to trust us they become more open to discussing their feelings and asking us questions about their condition”***(SNO**,** R32**,** 9 years of experience)**.

Fostering trust, providing compassionate communication, and offering emotional support are vital strategies that help patients navigate their fears and accept their reality, ensuring they feel secure and valued in their end-of-life journey.

### Theme 3: perceptions about End-of-Life care delivery

This theme explored nurses’ insights into the multifaceted aspects of providing care during patients’ final stages of life. These perceptions highlight the importance of specialized training, the acceptance of end-of-life as a natural process, the value of family involvement, respect for cultural and religious beliefs, and the emotional bonds formed with patients, shaping their approach to compassionate and holistic care.

#### Sub-theme 3:1 aspiring for additional training in End-of-Life care

The study participants expressed a strong desire for additional training in end-of-life care to improve their ability to support patients and families during these critical moments:

“*We need to keep updating our knowledge and skills to provide the best possible care to our patients. I am aware that learning new pain management techniques can significantly improve patient comfort. However*,* I am just a staff nurse and my expertise has primarily been acquired through practical experience. Therefore*,* I am considering enrolling in a course at Ghana College to further my knowledge and skills.*” **(SN**,** R12**,** 5 years of experience)**.

“ *During one training session*,* I learned about advanced counseling techniques and innovative therapies for anxiety and depression. These new methods allowed me to better support a patient who was experiencing severe anxiety as they neared the end of life. By applying these techniques*,* I could help ease their distress and provide a more compassionate care experience. I wish I had more experience in these areas; it would have made my work even more effective and less challenging.*” **(NO**,** R15**,** 6 years of experience)**.

Enhanced training programs can provide nurses with the necessary tools to manage complex end-of-life situations more effectively.

“*I studied communication in school*,* but it was more general and covered all aspects of patient communication*,* including verbal and non-verbal cues. Additionally*,* we only had one semester dedicated to palliative and gerontology. However*,* I believe this isn’t sufficient*,* especially given the complexities of caring for patients nearing the end of life*,* some of whom struggle with communication.”***(SNO**,** R10**,** 8 years of experience)**.

In conclusion, continuous professional development and specialized training in end-of-life care are essential for equipping nurses with the advanced skills and knowledge needed to navigate the complexities of patient communication, symptom management, and emotional support effectively.

#### Sub-theme 3.2 perceiving End-of-Life as inevitable and natural

Recognizing end-of-life as an inevitable and natural part encourages open communication about patients’ wishes and fosters a supportive environment where patients and families can find peace and acceptance during this critical time.The study participants emphasized the importance of perceiving end-of-life as an inevitable and natural part of life;

“*I recognize that death*,* growth*,* and illness are natural parts of life*,* and at some point*,* we may all face limited time. Understanding this*,* I approach patient care with the utmost respect*,* treating them as I would wish to be treated in their circumstances*” **(NO**,** R16**,** 7 years of experience)**.

“*We recognize that every beginning has an end*,* and this is true for life’s journey as well. Our goal is to ensure that patients understand their current stage and what they are experiencing. This understanding helps to reduce their anxiety levels and allows them to live their lives as normally as possible*.” **(SN**,** R21**,** 4 years of experience)**.

#### Sub-theme 3.3 nurses’ views on family engagement in End-of-Life care

Engagement of family in caregiving not only brings comfort to the patient but also helps family members feel included and supported during a difficult time. The study participants emphasized the importance of family involvement in providing compassionate and respectful care:

*" I remember caring for a patient whose family had a tradition of telling stories every time they visit. I have observed the patient really enjoys such moment with the family listening to the stories. By involving the family and arranging times for them to sing these hymns at the bedside*,* we created a comforting and familiar environment.*” **(NO**,** R20**,** 6 years of experience)**.

“*Here we encourage family involvement*,* I have observed family members take turns like we change shift as nurses. They have a way of making the patient laugh even if they do not feel like laughing. Hence*,* we ensure an atmosphere that helped the patient feel surrounded by love*” **(RN**,** R18**,** 8 years of experience)**.

Engaging families helps in creating a supportive environment where both patients and their loved ones feel cared for and valued.

“*There is a patient who has been hospitalized for over a year now. I’ve noticed that every weekend*,* her sons visit. One of them plays the guitar while the other sings for her. During these moments*,* they often shed tears and smile simultaneously. This touching routine usually helps the patient have a peaceful and long sleep afterward. We encourage this*.” **(SNO**,** R31**,** 8 years of experience).**

To conclude, embracing the natural cycle of life and fostering strong family involvement in end-of-life care creates a compassionate environment where patients find comfort and dignity, while families feel included, supported, and empowered to share meaningful moments with their loved ones.

#### Sub-theme 3.4 respecting patients’ cultural, social, and religious beliefs

Respecting patients’ cultural, social, and religious beliefs is crucial in end-of-life care. The study participants emphasized the importance of these aspects in delivering effective and compassionate care.;

“*Respecting a patient’s religious beliefs is crucial. In Ghana*,* some people believe in spiritual leaders known as Godfathers. Occasionally*,* these Godfathers visit to share prayers and words of hope. We allow these visits as long as the patient consents.*” **(NO**,** R19**,** 7 years of experience)**.

“*Some of our patients are Muslims and have specific prayer times throughout the day. We have arranged their care schedule to ensure it does not conflict with these times*,* except in emergencies. Our goal is to support their overall health while respecting their religious practices*” **(SNO**,** R31**,** 8 years of experience).**

“*We make an effort to understand each patient’s background and incorporate their beliefs into the care plan. This involves learning about their traditional practices and integrating these elements into our care approach. For example*,* we had a patient whose family practiced a specific end-of-life ritual involving the presence of certain family members and the chanting of traditional prayers. we permit it and allow them to exercise their belief and make sure it does not disturb other inmates*” **(SN**,** R28**,** 5 years of experience)**.

Respecting and integrating patients’ religious and cultural beliefs into their care plans fosters a supportive and inclusive environment, allowing patients to maintain their spiritual and cultural identity during end-of-life care.

#### Sub-theme 3.5 managing emotional connections with patients

Forming emotional connections with patients is an integral part of nursing care, especially in end-of-life care, but it can also be emotionally taxing. Nurses find ways to manage these connections while maintaining professional boundaries to ensure they can continue providing high-quality care without experiencing burnout. The study participants shared various strategies for managing these emotional connections effectively;

“ *In this hospital*,* we consider our patients to be our friends and family. End-of-life issues are challenging for both us and the patients*,* requiring us to be strong to provide the necessary emotional support. We strive to maintain a good relationship with our patients*,* encouraging them to see us as friends and to feel comfortable approaching us at any time. This is necessary because if they smile*,* we are also happy.*” **(NO**,** R23**,** 6 years of experience)**.

“*Building a relationship with patients is important*,* but it’s equally crucial to maintain professional boundaries to protect our own emotional health. For instance*,* I set aside time for self-care activities such as exercise and hobbies*,* and I make a conscious effort not to bring work-related stress home. I ensure that stress does not affect my wife and children. On weekends*,* when I am off duty*,* I sometimes share my emotions with my wife*,* and she encourages me and occasionally prays with me. She always reminds me*,* “You are strong*,* you got this*.” **(SN**,** R20**,** 5 years of experience)**.

Managing these emotional connections effectively helps nurses provide compassionate care without compromising their own well-being.

“*You cannot give what you do not have*,* By managing our emotional connections*,* we can provide compassionate care while also taking care of our own emotional well-being. For example*,* I regularly attend support groups for health care professionals to share experiences and coping strategies.* " **(SNO**,** R16**,** 8 years of experience)**.

Fostering emotional connections with patients while maintaining professional boundaries and prioritizing self-care enables nurses to provide compassionate care effectively without compromising their own emotional well-being.

**Discussion of Findings** This study investigated the specific end-of-life care needs of patients and their families while identifying strategies to enhance nursing practices in selected healthcare facilities in Accra, Ghana. Nurses demonstrated a strong commitment to providing dignity and comfort through family involvement, compassionate pain and symptom management, and navigating difficult end-of-life conversations. They created positive memories by celebrating special occasions such as final birthdays and anniversaries and enabling patients to spend meaningful time with loved ones. For mobile patients, outings and recreational activities outside the facility enriched their experiences, fostering emotional well-being and connection. To sustain this high level of care, nurses require acknowledgment, motivation, and incentives, alongside adequate staffing and specialized training in comfort and pain management. Collaboration with volunteers to oversee outings and therapy sessions for patients struggling with coping challenges also proved beneficial. These efforts underline the importance of personalized, patient-centered care in improving quality of life at the end of life, aligning with prior research on the role of memory creation and recreational activities in enhancing end-of-life experiences [33–36].

The study underscored the importance of respecting and upholding ethical values in end-of-life care, focusing on patient autonomy, managing mixed reactions such as gratitude and discontent, and supporting patients in accepting the reality of their condition. Challenges arose when respecting patient autonomy conflicted with medical recommendations, particularly when patients’ choices resulted in less favorable outcomes. Nonetheless, nurses emphasized the importance of advocating for patient desires, even when they diverged from clinical advice, ensuring informed decision-making while maintaining autonomy. Ethical care necessitated strategies like patient education, shared decision-making, and advance care planning, aligning with existing literature on the complexities of medical decision-making [37, 38]. Additionally, nurses demonstrated resilience by embracing words of gratitude while effectively managing patient dissatisfaction, ensuring that ethical values were upheld and patient needs were met. Building trust and fostering open communication were pivotal in delivering compassionate end-of-life care, as supported by research highlighting these practices as essential for quality care [38, 39].

Nurses held several perceptions about end-of-life care, including viewing death as a natural part of life, recognizing the vital role of family involvement, respecting cultural, social, and religious practices, and maintaining emotional connections with patients. These perspectives enable health care providers to prioritize peace and comfort in patients’ final days, offering dignified and compassionate care that eases anxiety, strengthens family bonds, and honors individual beliefs. Such approaches preserve patient dignity, reduce psychological distress, and improve overall end-of-life care quality, as supported by evidence [40]. In Ghana, spiritual leaders, known as Godfathers, often visit to offer prayers and hope, while Muslim patients adhere to specific prayer times. Nurses accommodated these practices, even if they didn’t personally subscribe to them. Integrating family meetings and incorporating cultural and religious practices have been shown to enhance satisfaction and emotional well-being [10, 41, 42]. The study highlighted the importance of prioritizing nurses’ self-care while ensuring ongoing training and education in palliative and end-of-life care. Addressing providers’ well-being, along with regular updates on clinical methods and best practices, enhances patient outcomes, addresses care challenges, and improves the quality of life for patients in their final stages [39, 43].

### Strengths and limitation

This study provides valuable insights into nurses’ experiences with end-of-life care in Ghana but has notable limitations. It focused exclusively on nurses, excluding perspectives from other critical members of the palliative care team, such as physicians, social workers, spiritual leaders, and pharmacists, potentially limiting a comprehensive understanding of the multidisciplinary dynamics essential for effective end-of-life care. However, the study intentionally emphasized the unique roles of nurses as primary caregivers. Additionally, some contextual cultural and religious findings may not fully translate to developed countries with distinct healthcare systems and resources due to differences in societal norms and healthcare infrastructure. Finally, the use of descriptive phenomenology with inductive analysis, while effective in capturing participants’ lived experiences, limits the study by not utilizing an established theoretical framework, which could have provided deeper interpretive and conceptual insights.

## Conclusion

The study highlighted that nurses prioritized dignity and comfort in care, upheld patients’ ethical values, and maintained positive perceptions of end-of-life care. They demonstrated compassion by alleviating pain and symptoms, addressed the challenges of sensitive end-of-life conversations, and actively ensured family involvement in care. These findings underscore the need for enhanced support and training for nurses in low-resource settings to improve the quality of end-of-life care. Future research could explore the experiences of the entire palliative care team and investigate interventions to strengthen palliative care in similar resource-limited contexts.

## Data Availability

All supporting data have been provided within the manuscript.
